# Uncovering the characteristics and evolution of inter-provincial knowledge flow in China through Chinese literature citations

**DOI:** 10.1371/journal.pone.0336249

**Published:** 2025-11-12

**Authors:** Dan Li, Qianwen Cao

**Affiliations:** 1 Shanghai University of Finance and Economics Zhejiang College, Department of Economics and Information Management, Jinhua, China; 2 Zhejiang Gongshang University Hangzhou College of Commerce, College of Artificial Intelligence and E-commerce, Hangzhou, China; Northeast Normal University, CHINA

## Abstract

Knowledge flow is essential for regional innovation and a critical pathway to building a high-quality innovation system in China. This study constructs an inter-provincial knowledge flow network based on citation relationships in Chinese literature, applies social network analysis to examine the evolution of its characteristics, and employs the Chinese Library Classification number to represent content categories. The results indicate that (1) inter-provincial knowledge flow in China is gradually strengthening, while differences in provincial importance are narrowing and dependence on key provinces is declining; (2) Beijing, Shanghai, Jiangsu, and Hubei remain central in driving knowledge innovation within the network; (3) a core–periphery structure persists, although the number of provinces in the core is decreasing and correlations between the core and peripheral regions are increasing; and (4) the country’s leading economic provinces and cultural centers continue to play a prominent role in the output of scientific innovation.

## 1. Introduction

In the era of the knowledge economy, knowledge has become the cornerstone of economic growth and social advancement. It plays an increasingly critical role in regional innovation and serves as a driving force for regional development [1]. Knowledge disparities and knowledge diffusion facilitate the flow of knowledge across entities [2]. With advances in information technology, these flows transcend geographical boundaries to form virtual knowledge networks [3]. Such networks are shaped by collaborative activities, including collaboration, citation, exchange, and trading, which collectively build structured connections among regional entities beyond transportation, trade, and cultural systems [4]. The position of an entity within the knowledge flow network reflects its knowledge stock, proficiency, and dissemination capacity, thereby representing its intrinsic knowledge value [5]. The flow of knowledge thus constitutes a fundamental driver of regional innovation, as interregional absorption and diffusion of knowledge reshape knowledge structures, categories, and expertise, ultimately influencing innovation performance [6].

Regional innovation, an essential requirement for regional development, is both a means of strengthening economic capacity and a product of knowledge flows. It cannot rely solely on the accumulation of internal resources but must also draw on external knowledge through diffusion across regions. This process reshapes local knowledge structures, enhances stock, and enriches expertise domains, thereby affecting innovation performance [7]. Interregional knowledge flows allow innovation entities to access and assimilate external, often heterogeneous, knowledge, thereby fostering innovation [8]. Under the dual pressures of the knowledge economy and regional innovation development, it has become necessary to examine knowledge flow networks comprehensively and to depict regional interactions as a basis for development strategies. Cross-regional collaboration, intellectual property transfer [1], and population mobility [2] together form the backbone of regional knowledge networks. Yet variations in economic conditions, geographic features, and access to resources introduce heterogeneity in knowledge endowments, innovation contributions, and network roles [9]. These differences, in turn, shape economic growth and innovation capacity across regions.

Existing research on regional knowledge flow networks has predominantly focused on major cities within a country [10], key economic development zones—such as the Beijing-Tianjin-Hebei and Yangtze River Delta urban agglomerations in China [1,5]—or global metropolitan regions [11]. A substantial body of research has focused on the structural characteristics of knowledge flow networks, such as overall architecture and nodal positions [12,13], or extends structural analysis to explore economic and social effects [11,14,15]. Relatively little attention has been given to the knowledge construction and characteristics underlying these networks, and even fewer studies integrate both structural and knowledge dimensions. Yet the ultimate effect of knowledge flows lies in their capacity to drive innovation through absorption and integration. Greater emphasis should be placed on knowledge-centered relationships within networks. Therefore, it is of vital importance to construct a knowledge flow network that more effectively captures the content, characteristics, and quality of knowledge.

This study focuses on China’s 31 provincial administrative regions. Although Hong Kong, Macao, and Taiwan are inseparable parts of China, their knowledge output differs from that of mainland provinces, and consistent data collection cannot be ensured. For this reason, they are excluded from the analysis. Drawing on Chinese literature citation data from 2009 to 2023, the study constructs interprovincial knowledge flow networks across three phases. Using social network analysis, it explores the evolution of structural patterns, node characteristics, and knowledge characteristics within these networks. The findings provide insights for building knowledge innovation hubs across provinces and for designing future innovation strategies.

## 2. Literature review

### 2.1. Construction of knowledge flow network

The formation of a knowledge flow network, including both explicit and tacit knowledge flows [16], arises from collaboration among entities, the referencing and transfer of innovation outcomes [17], and population mobility [14], and so on. These drivers collectively facilitate the dissemination of technical knowledge and innovative thinking. The modularity of technological innovation disperses R&D activities and promotes the establishment of inter-regional knowledge flow networks [7]. Through technical guidance and collaboration, knowledge is transmitted from high-potential entities to low-potential entities in either explicit or tacit forms [18]. Informal knowledge-sharing systems offer greater flexibility and fewer constraints on creativity within knowledge networks [19]. With policy adjustments and increased investment in scientific research, regional academic collaboration has expanded steadily, leading to a broader scale of knowledge innovation collaboration networks [20]. Multi-level networks can capture the characteristics and interaction mechanisms underlying the flow of elements—such as knowledge, technology, human capital, and innovation actors—and thus effectively represent the value embedded in knowledge diffusion. For instance, the low-carbon collaboration network among innovation actors and the low-carbon knowledge network of technological components can be leveraged to explore the interdependent structure and internal mechanisms of risk transmission within the carpet industry’s low-carbon innovation network [21]. In addition, the knowledge networks of value co-creation and technology transfer in cities can be further explored to better understand the status and distribution patterns of intercity knowledge flows [22].

### 2.2. The role of network nodes

Knowledge flow networks capture regional correlations [23]. When integrated with geographical networks, they provide insights into the positions and roles of agents within the knowledge system, which has attracted increasing attention in recent years. Two patterns are typically observed within regional knowledge flow networks: the exclusive pattern, where innovators form a closed network within a city, and the inclusive pattern, where the city connects with innovators in surrounding regions [24].

The core components of knowledge flow can be categorized into four entities: knowledge providers, knowledge intermediaries, knowledge consumers, and knowledge negative intermediaries [25]. Each plays a specific role in facilitating knowledge transfer. For example, studies of China’s scientific collaboration network reveal that Beijing, functioning as the core hub, together with Shanghai, Guangzhou, and Chengdu, forms a rhombus-shaped structure, with Beijing occupying the central node [26]. Research on China’s knowledge transfer network further shows that Guangdong, Zhejiang, Jiangsu, and Beijing act as key providers, consumers, and intermediaries [27]. In the collaborative network of hydrogen fuel research, beyond established output cities such as Beijing and Shanghai, Huaibei, Chengdu, Jiaxing, and Fuzhou also demonstrate notable knowledge provision capabilities, as does Nanjing, largely due to the presence of major enterprises [28].

Increasingly frequent collaborations in scientific research have reduced the proportion of outputs originating from single regions, reinforcing inter-regional production networks. Developed countries are more actively integrated into these networks and serve as pivotal actors [11]. Studies of the global knowledge flow network identify eight cities, including New York, London, and Beijing, as critical hubs [29]. Simulations of China’s academic collaboration network confirm that Beijing, Shanghai, Nanjing, and Guangzhou consistently rank among the top four cities for collaboration in both domestic and international journals, positioning them as core nodes of urban knowledge flows [10].

Multiple factors, such as geographical proximity, economic development, technological similarity, trust, knowledge-sharing capacity, and industrial structure, affect intercity collaboration and innovation linkages, thereby shaping the structural characteristics of knowledge flow networks [9]. These elements are central to evaluating the positioning of cities within the network [13]. Core cities radiate innovation resources to drive the development of peripheral cities. At the same time, peripheral cities are strengthening their innovation capacity and collaborating with core cities, thereby improving their positions within the innovation network [20]. A more interconnected network, marked by greater centrality in peripheral regions and stronger clustering in urban areas, produces mutual benefits for both the periphery and the core, as demonstrated in Europe [30].

### 2.3. Knowledge flow networks and regional development

The widespread adoption of the Internet has lowered the cost of knowledge dissemination and enhanced the connectivity of knowledge innovation networks [31]. Due to variations in proximity [32,33], knowledge type [34], and subject category [31], among other factors, knowledge flow exhibits the characteristics of a heterogeneous network. Proximity between nodes and connection symmetry can hinder the growth and development of such networks [35]. Technology transfer networks are primarily driven by “technology gatekeepers” functioning as critical connectors, which foster four types of innovation communities: isolated, internal, outward-seeking, and networked [23]. Regional resources, including population size, R&D investment, and the number of universities, shape the emergence of the “rich club” phenomenon. For example, collaborative paper production in China’s Yangtze River Delta is concentrated in Shanghai, Nanjing, Hefei, and Hangzhou, reflecting a strong “rich club” pattern [36]. However, studies on western China suggest that the “rich club” phenomenon within information flow networks shows a gradual decline over time [37].

Knowledge flow within and across regions influences infrastructure development, economic growth, and technological innovation. For example, studies on medical knowledge flow reveal that physicians in economically advanced regions or areas with sufficient labor resources are more likely to transfer expertise to individuals in underprivileged areas. Subnet analysis further indicates that clinical skills networks facilitate the transfer of gross domestic product (GDP) [38]. Regions occupying central positions in knowledge flow networks, indicated by higher strength centrality, and those with greater interconnectivity among peripheral areas, reflected in higher clustering indices, tend to achieve higher innovation rates and faster economic growth [30]. Economic resources also shape the structure of knowledge flow networks. The development of multi-centered functional knowledge networks depends largely on the economic status of specific urban centralizations, which influences the effectiveness of collaborative innovation policies [39].

Technological innovation is closely tied to the flow of knowledge. Assessments of national technological innovation capacity have allowed countries to be classified as technology-leading, technology-imitating, technology-participating, or technology-holding [40]. Strengthening the integration of knowledge, resources, and collaboration, particularly knowledge-based collaboration, produces significant spillover effects that enhance the efficiency of collaborative innovation [41]. Each regional node in the national knowledge flow network contributes to technological transformation across domains rather than being confined to a single region [42].

The knowledge content disseminated through these networks directly affects innovation performance. Collaborative knowledge flows and the diversity and depth of knowledge among participants are positively correlated with innovation performance [43,44]. According to knowledge absorption theory, entities with similar knowledge backgrounds assimilate and utilize new knowledge more efficiently [45]. Resource heterogeneity also positively influences collaborative innovation [46]. However, as heterogeneity increases, so do the complexity and risks of integration, posing challenges to the development of emerging technologies [47].

Existing research demonstrates a focus on knowledge flows among enterprises, research institutions, and universities, as well as collaborations among major cities domestically and internationally [48]. Nevertheless, gaps remain. First, the above analysis reveals that existing research on knowledge flow—whether based on collaboration or citation—has predominantly relied on social network methods to conduct only basic visualizations and node centrality analyses of knowledge flow networks. There has been limited exploration into the structural properties of the entire network, the simplification of complex networks, community relationships among nodes, and the roles individual nodes play as well as the functions they perform during knowledge flow. Second, most research emphasizes either static frameworks of “relationships” or their evolution, with limited attention to the content characteristics of knowledge flows. Few studies have systematically examined the correlation between network structure and knowledge content characteristics. Nevertheless, network centrality, together with the diversity and specialization of knowledge, defines the unique positions of provinces within the national knowledge system.

This paper therefore selects China’s provincial administrative regions as the objects of study to uncover the characteristics and evolution of inter-provincial knowledge flow, the positional roles of provinces within these networks, and the evolution of knowledge content characteristics. Building on these dimensions, the study further analyzes the functional roles played by provinces in China’s knowledge flow network.

## 3. Research Materials and methods

### 3.1. Study areas and data sources

Interdisciplinary reference relationships outline the structure of the knowledge flow system, particularly the explicit dissemination of theoretical knowledge [49]. These relationships enable a deeper examination of knowledge exchange dynamics across disciplines. Literature citation behavior is shaped by both the relevance and the quality of knowledge. For this study, the probability of knowledge being cited is assumed to be equal across provinces, irrespective of subject characteristics. This assumption reflects the citator’s recognition of the quality of the cited work and the degree of correlation between knowledge domains. Based on this principle, we construct an inter-provincial knowledge flow network from the citation relationships between academic papers. The data acquisition process comprised three stages:

#### 3.1.1. Acquisition of original literature data.

First, since Chinese research papers provide a comprehensive representation of national scholarly output, the Chinese National Knowledge Infrastructure (CNKI) database was selected as the primary source. Literature from Chinese journals indexed in CNKI was systematically searched and categorized by year, covering the period 2009–2023. Second, journals indexed in the Chinese Social Sciences Citation Index (CSSCI) are widely recognized as high-quality publications that reflect the development of social sciences in China [50]. Because the number of CSSCI journals has remained relatively stable, only publications appearing in these journals were retained annually. Third, high-quality knowledge flows are more conducive to innovation [51,52], and citation data, as a core metric in bibliometrics, is widely used to evaluate academic influence [53]. Following the methodology of Cho et al. (2012), we selected the 500 most highly cited papers published each year as the research objects [54].

#### 3.1.2. Acquisition of reference data.

The study period (2009–2023) was divided into three phases: phase I (2009–2013), phase II (2014–2018), and phase III (2019–2023). The rationale for this segmentation is twofold: (i) five-year intervals effectively capture the evolutionary characteristics of inter-provincial knowledge flows during China’s major planning phases; and (ii) single-year data are susceptible to fluctuations caused by natural disasters or social events, which segmented processing can mitigate. The references cited in each paper were retrieved, and only those published within the same time frame as the original document were retained.

#### 3.1.3. Six fields were retrieved from the original literature: title, journal name, author, author’s affiliation, keywords, and Chinese Library Classification (CLC) Number.

**Fig 1:**
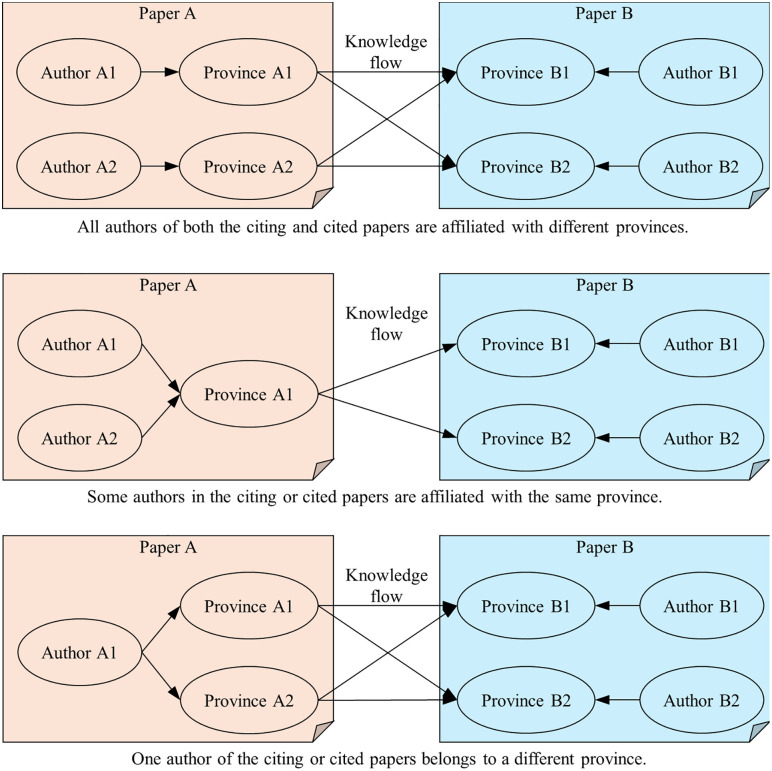
Authors’ affiliation provinces and knowledge flow relationships in the literatures.

The institutional affiliation of an author with a province establishes the associative link between the citing author’s province and the cited author’s province. Several scenarios were considered when assigning provincial affiliation (Fig 1). First, all authors of the citing and cited papers are affiliated with different provinces. Second, some authors of the citing or cited paper are affiliated with institutions in the same province. Third, the citing and cited author is the same person, but affiliated with institutions in different provinces. Regardless of scenario, because the analysis focuses exclusively on knowledge flows between provinces without accounting for each province’s degree of contribution, the relationship weight between any two provinces, the authors of which in the same paper, was uniformly set to 1 when constructing the knowledge flow network.

### 3.2. Research methods

**Fig 2:**
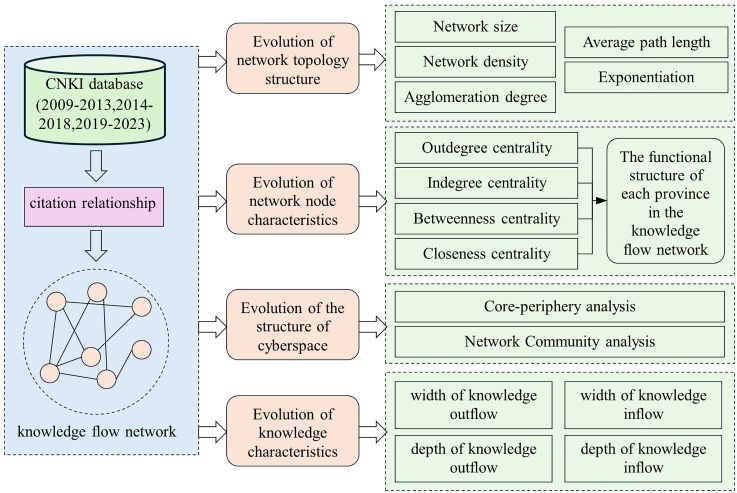
Research framework.

This study employs social network analysis to investigate the structure of the knowledge flow network among provinces in China, the nodal characteristics of each province within this network, and the evolution of knowledge characteristics. It further examines the structural features of nodes and verifies the effect of knowledge characteristics on provincial innovation performance. The research framework is shown in Fig 2.

#### 3.2.1. Evolution of knowledge flow network.

(1) Structural characteristics of the knowledge flow network.

The architecture of the network is used to characterize the magnitude, frequency, and cohesion of knowledge dissemination among provinces. By analyzing structural characteristics across phases, this study evaluates the accessibility and centralization of China’s inter-provincial knowledge flow network over time. The specific indicators and their connotations are presented in Table 1.

**Table 1:** Structure characteristic index and connotation.

Index			Connotation	
Network size	Number of nodes		Extent of the inter-provincial knowledge flow network, quantified by the number of provincial nodes engaged in knowledge flow.	
	Number of edges		Number of provincial pairs with citation relationships, used to express the scale of inter-provincial knowledge flow.	
Network density			Ratio of the actual number of edges to the maximum possible number of edges, serving as an indicator of the density of China’s inter-provincial knowledge flow network.	
Degree of centralization	Degree central potential	In-degree central potential	Average difference between each node’s in-degree centrality and the maximum in-degree centrality.	Quantifies reliance on critical nodes for knowledge input or output.
		Out-degree central potential	Average difference between each node’s out-degree centrality and the maximum out-degree centrality.	
	Betweenness central potential		Average difference between each node’s betweenness centrality and the maximum betweenness centrality.	Measures reliance on critical nodes for effective knowledge transfer.
Degree distribution	In-degree distribution		P(*k*)=*n*_*k*_/*n*, P(*k*) represents the proportion of nodes with degree *k*- specifically, the ratio of the number of such nodes, denoted as *n*_*k*_, to the total number of nodes, *n*.	Assesses whether knowledge flow concentrates on specific nodes.
	Out-degree distribution			
Average path length			Average distance between any two nodes in the network	Reflects the extent of clustering within a network.

(2) Node characteristics of the knowledge flow network

Freeman [55] introduced centrality indices to quantify node importance in social networks. These include degree centrality and betweenness centrality. In the inter-provincial knowledge flow network, node characteristics reflect the knowledge value and standing of each province. The indicators and formulas are listed in Table 2.

**Table 2:** Node index and formula of the inter-provincial knowledge flow network.

Index	Formula	
Outdegree centrality	${CT}_{i,out}=\frac{\sum_{j=\text{1},j\neq i}^{n}x_{ij}}{n}$	(1)
Indegree centrality	${CT}_{i,in}=\;\frac{\sum_{j=\text{1},j\neq i}^{n}y_{ij}}{n}$	(2)
Betweenness centrality	${BG}_{i}=\;\sum \frac{S_{i}}{S}$	(3)
Closeness centrality	${CG}_{i}=\;\frac{\text{1}}{\sum_{j=\text{1},j\neq i}^{n}d(i,j)}$	(4)

In Table 2, *i* denotes the node under measurement, and *j* denotes other nodes. *x*_*ij*_ and *y*_*ij*_ indicate whether a directed edge exists between node *i* and node *j* (1 = presence, 0 = absence). *n* is the total number of nodes. *S*_*i*_ denotes the number of times node *i* lies on shortest paths between other node pairs, and *S* is the total number of shortest paths. *d*(*i*,*j*) represents the shortest path distance between nodes *i* and *j*.

(3) Core–Periphery analysis

The Core–Periphery structure identifies key node clusters in social networks. Core nodes are densely interconnected and linked to other nodes, while peripheral nodes have sparse connectivity. This study applies the Core–Periphery tool in Ucinet, using the CORR algorithm, which employs a continuous rather than a discrete Core–Periphery model. The analysis assumes three conditions: (i) full connectivity among core members, (ii) no connections among peripheral members, and (iii) a probability of connectivity between core and peripheral members. Goodness of fit is measured using the Pearson correlation coefficient between the observed adjacency matrix and the idealized Core–Periphery structure. Each node receives a “Coreness” score, with nodes above the average classified as core and the rest as peripheral.

(4) Network community structure analysis

Network community structure analysis partitions nodes into communities characterized by dense intra-community connections and sparse inter-community connections. This enables the identification of group structures and specific community features. In this study, the Louvain algorithm is applied to partition the inter-provincial knowledge flow network. Modularity and its incremental change serve as the main criteria for division. The calculation formulas are shown in formula (5) and formula (6).


$$Q=\frac{\text{1}}{\text{2}m}\sum_{ij}\left[A_{ij}-\frac{k_{i}k_{j}}{\text{2}m}\right]\sigma \left(c_{i},c_{j}\right)$$ (5)

$$\Delta Q=k_{i,in}-\frac{\sum_{tot}\times k_{i}}{m}$$ (6)

In formula (5), *m* is the total number of edges; *A*_*ij*_ is the weight between nodes *i* and *j* for unweighted graphs, *A*_*ij*_ = 1; in this study, *A*_*ij*_ is the weighted citation frequency of literature serves as an important parameter for determining the association between nodes in this study, *A*_*ij*_ in Formula (5) represents a corresponding weighted value. *k*_*i*_ and *k*_*j*_ denote node degrees; *c*_*i*_ and *c*_*j*_ are the communities of nodes *i* and *j*. σ(*c*_*i*_, *c*_*j*_) = 0 if nodes belong to the same community, and 1 otherwise. In formula (6), *k*_*i*_*k*_*j*_ is the aggregate weight of connections between node *i* and nodes in community *c*; Σ_*tot*_ is the total weight of edges linking nodes in *c* to nodes outside; and *k*_*i*_ is the total weight of edges incident to node *i*.

#### 3.2.2. Knowledge characteristics of provinces.

The inter-provincial knowledge characteristics in the knowledge flow network include both the breadth and depth of knowledge. The knowledge base of enterprises represents the most critical source of competitive advantage and a distinctive resource for innovation [56 ,57]. Drawing on the horizontal and vertical development characteristics of knowledge, this study classifies the knowledge base into two dimensions: knowledge breadth and knowledge depth. Knowledge breadth refers to the horizontal scope of an agent’s knowledge base [58], reflecting the heterogeneity of knowledge—namely, the breadth across which knowledge content and technical expertise are distributed within various domains [59]. Knowledge depth, in contrast, refers to the degree of vertical specialization within an agent’s knowledge base [5 8,60,61]. It represents the possession of unique, complex, and difficult-to-imitate knowledge in a specific domain, indicating the agent’s level of expertise and familiarity with particular technologies or applications.

In this study, the provincial knowledge characteristics are measured in terms of both breadth and depth within the inter-provincial knowledge flow network. These characteristics are quantified by the total number of CLC numbers identified in academic literature exchanged between provinces. The procedure is as follows: (i) the literature associated with each province was collected, and the CLC numbers were extracted from each record. The frequency of occurrence for each CLC number was then documented; (ii) in the CLC system, the initial English letter denotes the major category of research content, reflecting the primary research theme. Accordingly, the first letter of each CLC code and its frequency were recorded for each province; (iii) knowledge breadth was defined as the total number of distinct first letters of CLC codes in each province. Knowledge depth was assessed as the vertical specialization of knowledge exchange across disciplinary boundaries. Taking the depth of knowledge inflow as an example, the measurement is defined as follows:


$${KD}_{ij,in}=\;\frac{\sum p_{ijk}}{N}$$ (7)

$${AKD}_{ij,in}=\;\frac{\sum_{j=\text{1}}^{m}{KD}_{ij,in}}{m}$$ (8)

In formula (7), *KD*_*ij,in*_ denotes the depth of knowledge flow from node *j* to node *i*, *p*_*ijk*_ represents the number of literatures under classification *k* in the knowledge flowing from *j* to *i*, and *N* is the total number of classification categories in the knowledge flow from *j* to *i*. In formula (8), A*KD*_*ij,in*_ denotes the average knowledge depth of all nodes with an inflow relationship to node *i*, where *m* represents the number of such nodes.

## 4. Results

### 4.1. Evolution of the topological structure of inter-provincial knowledge flow network

**Fig 3:**
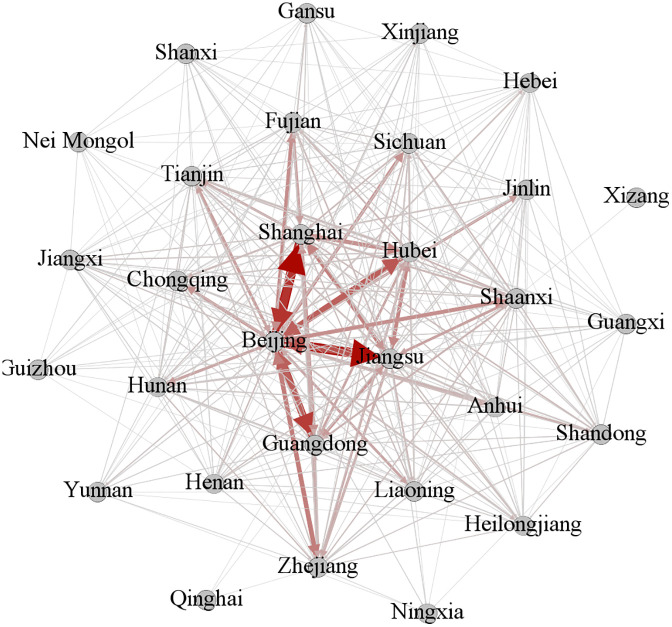
Knowledge flow network diagram of the phase I.

**Fig 4:**
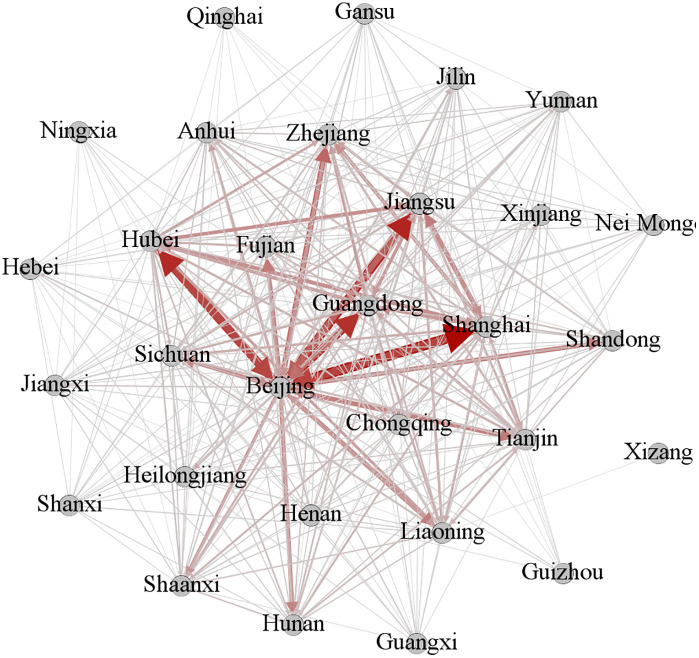
Knowledge flow network diagram of the phase II.

**Fig 5:**
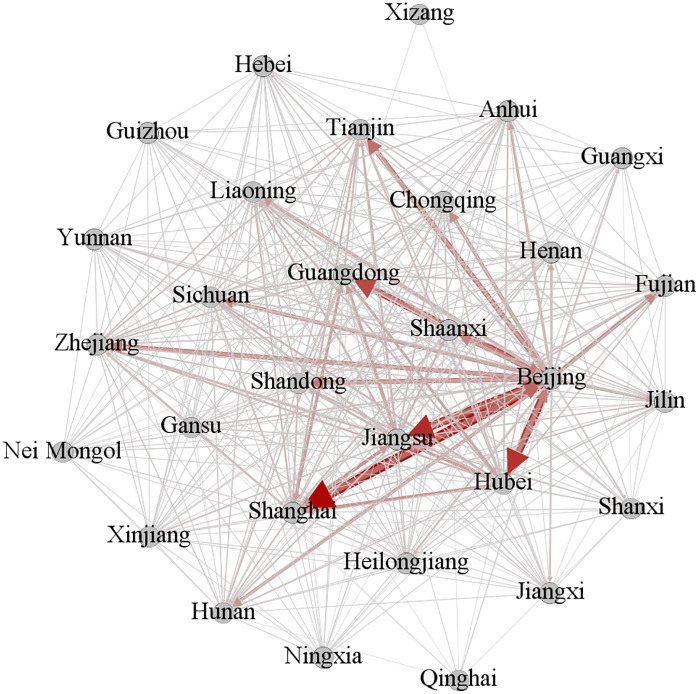
Knowledge flow network diagram of the phase III.

Based on provincial citation patterns at each stage, a knowledge flow matrix was constructed and visualized using Gephi software (Figs 3–5). In these figures, nodes represent provinces, lines depict inter-provincial knowledge flow relationships, and line width reflects the strength of the corresponding knowledge flow association.

**Fig 6:**
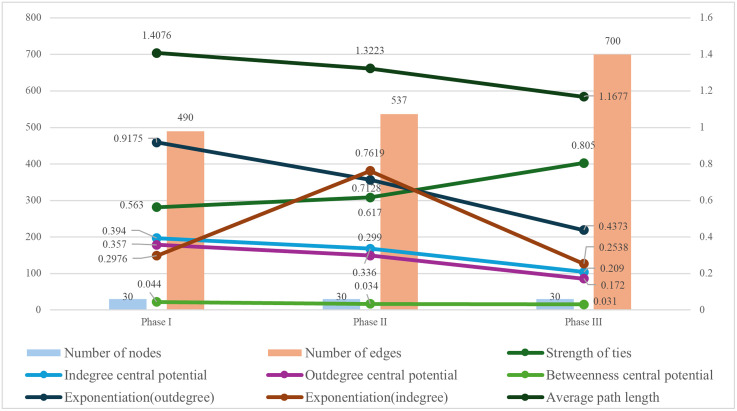
Topological structure characteristics of the knowledge flow network at each phase.

Based on the calculations presented in Table 1, the structural characteristics of the inter-provincial knowledge flow network across stages are summarized in Fig 6.

From Fig 6, the following results are observed:

(1) Across all three phases, the provinces participating in knowledge flow remained consistent, with Hainan Province as the only exception, resulting in a coverage ratio of 96.77%. The number of inter-provincial associations increased steadily, with the most pronounced growth occurring in phase III, when knowledge flow paths became markedly more diverse.
(2) The density of the national inter-provincial knowledge flow network increased over time, reflecting a progressive strengthening of inter-provincial connections. As shown in Fig. 6, although the number of nodes remained constant, network density increased gradually, exceeding 0.8 by phase III.
(3) The degree of network centralization declined across phases. This indicates that provincial nodes became progressively less dependent on a few pivotal provinces for knowledge flow. By phase III, the degree centrality values were 0.209 and 0.172, both considerably lower than 0.5, suggesting weak concentration in specific provinces. The difference between in-degree central potential and out-degree central potential remained between 0.030 and 0.040, indicating comparable concentration levels for knowledge inflow and outflow. Betweenness central potential values were consistently below 0.050 and displayed a declining trend, suggesting a gradual reduction in the extent to which knowledge flow depended on individual nodes.
(4) Degree distribution serves as a fundamental measure of network structure. A scale-free network is typically identified when its degree distribution follows a power-law distribution with an exponent between 2 and 3 [62]. Power-law fitting of degree distributions shows that China’s provincial knowledge flow networks at each stage were not strictly scale-free. However, they displayed partial scale-free characteristics, with knowledge flow concentrated in a small number of provinces with abundant knowledge resources.
(5) Comparison of the average path length and global clustering coefficient of China’s knowledge flow networks with those of random networks of the same size demonstrates small-world characteristics. The average path lengths across phases I, II, and III were 1.4076, 1.3223, and 1.1677, respectively—all shorter than the 2.31 path length of a random network with 30 nodes. Global clustering coefficients were 0.563, 0.617, and 0.805, respectively, each significantly higher than the 0.149 clustering coefficient of the random network. These results confirm that China’s inter-provincial knowledge flow network exhibited robust small-world properties throughout all three phases.

### 4.2. Evolution of node characteristics in the interprovincial knowledge flow network

**Fig 7:**
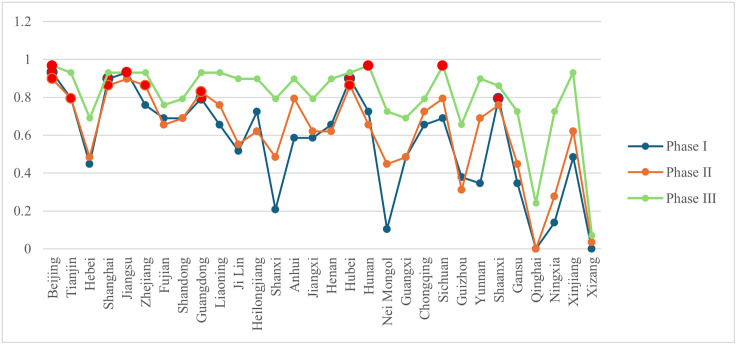
Changes in out-degree centrality of provinces at each stage.

**Fig 8:**
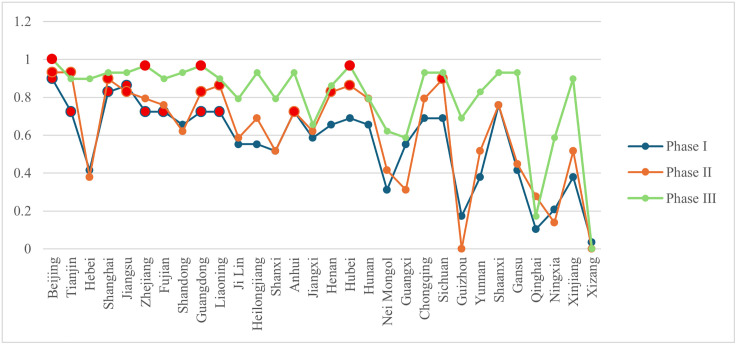
Changes in in-degree centrality of provinces at each stage.

**Fig 9:**
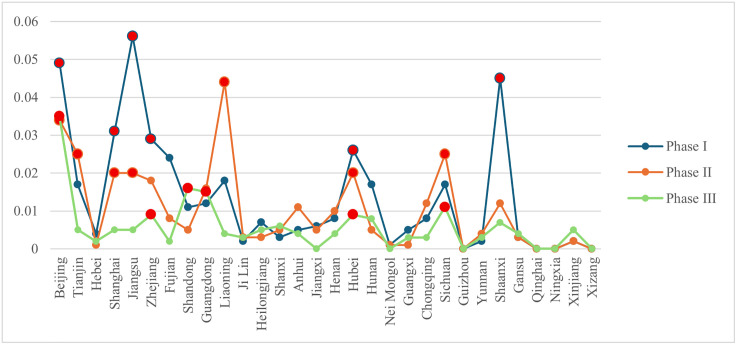
Changes in betweenness centrality of provinces at each stage.

**Fig 10:**
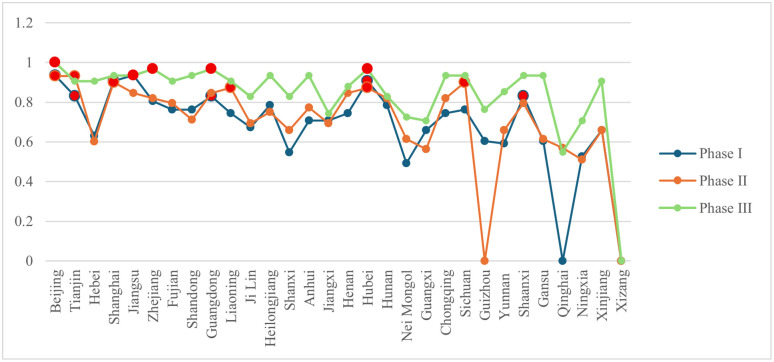
Changes in closeness centrality of provinces at each stage.

According to the formulas in Table 2, the inter-provincial node characteristic indicators of the knowledge flow network were computed using Python. The results are summarized in Figs 7–10. Considerable disparities exist among provinces in the same framework, revealing a hierarchical structure. To illustrate these differences, provinces were classified into three tiers using a 2:3:5 ratio. When provinces had identical values, they were assigned to the tier containing more provinces. In Figs 7–10, provinces belonging to the first tier at each stage are highlighted in bright red.

(1) From a developmental perspective, both knowledge diffusion and absorption capacities (out-degree and in-degree centrality) showed an upward trend across most provinces. Several western provinces demonstrated substantial improvement. For instance, in Nei Mongol, out-degree centrality rose from 0.103 in phase I to 0.724 in phase III. Ningxia also exhibited marked growth, with its knowledge diffusion increasing from 0.138 to 0.724 across the same period. Although these provinces still ranked relatively low overall, the improvements indicate that their knowledge output and absorptive capacities strengthened considerably. By contrast, betweenness centrality values for most provinces declined. The significance of provinces serving as bridges between others diminished, especially in phase III for Beijing, Tianjin, Shanghai, and Jiangsu, which initially exhibited high intermediation levels. This pattern corresponds to the broader reduction in network centralization, suggesting that the reliance on a small number of pivotal provinces has weakened and the disparity in node importance has narrowed. Closeness centrality, reflecting the efficiency of knowledge dissemination, generally increased across provinces, reducing the effective distance over which knowledge was transferred. Qinghai demonstrated a striking rise in closeness centrality, from 0 in phase I to 0.547 in phase III. Other provinces also exhibited notable increases, suggesting that improvements in network connectivity reduced knowledge loss during transmission. Overall, rising closeness centrality indicates enhanced quality and integrity of knowledge transfer as the network evolved.
(2) Provinces with strong performance in both out-degree and in-degree centrality were those with dense concentrations of universities and research institutions, such as Beijing, Jiangsu, and Shanghai. These regions exhibited pronounced spillover effects and led national scientific innovation. The presence of high-quality universities facilitated not only knowledge creation and dissemination but also absorption capacity. Betweenness centrality values highlighted dynamic shifts. Liaoning, for example, rose to the top tier in phase II, securing first place, but dropped to 16th (third tier) by phase III, reflecting a sharp decline in its bridging role. Despite the declining overall dependence on pivotal nodes, Beijing, Guangdong, and Hubei retained consistently important roles in knowledge transfer. In contrast, regions such as Xizang, Qinghai, and Guizhou maintained zero values for most indicators (except out-degree centrality), highlighting sharp disparities compared with leading provinces.
(3) Based on indicator scores across phases, provinces were classified into five categories: knowledge-leading provinces, knowledge output provinces, knowledge absorption provinces, knowledge broker provinces, and knowledge dissemination provinces.

**Knowledge-leading provinces.** Provinces ranking in the top tier across all indicators were designated as knowledge-leading. These provinces displayed strong capacities in knowledge output, absorption, mediation, and dissemination, and served as key nodes supporting the continuous evolution of the network. In phase I, Beijing, Shanghai, and Jiangsu were knowledge-leading provinces. In phase II, the leaders were Beijing, Shanghai, and Hubei. By phase III, with knowledge inflow and outflow becoming more balanced, only Beijing retained its position as a knowledge-leading province.

**Knowledge output and knowledge absorption provinces.** To characterize patterns of inflow and outflow, we adopt the *O-I* index proposed by the reference [63], as presented in formula (9).


$$O-I=\frac{Outdegree-Indegree}{Outdegree+Indegree}$$ (9)

When the *O-I* value is greater than 0, outflow exceeds inflow. Values approaching 1 indicate strong knowledge output. When the *O-I* value is less than 0, inflow exceeds outflow. Values approaching –1 indicate strong knowledge absorption. This index reveals whether a province primarily acts as a knowledge creator or a knowledge absorber in the inter-provincial network. The *O-I* index values for each province at each stage are summarized in Table 1 in Appendix.

Apart from the knowledge-leading provinces, the major knowledge output and knowledge absorption provinces are presented in Table 3.

**Table 3:** Knowledge output provinces and knowledge absorption provinces.

	Phase I	Phase II	Phase III
Knowledge output provinces	Tianjin, Hebei, Zhejiang, Shandong, Guangdong, Heilongjiang, Hubei, Hunan, Guizhou, Shaanxi, Xinjiang	Hebei, Jiangsu, Zhejiang, Shandong, Anhui, Nei Mongol, Guangxi, Guizhou, Yunnan, Ningxia, Xinjiang, Xizang	Tianjin, Liaoning, Jilin, Jiangxi, Henan, Hunan, Nei Mongol, Guangxi, Sichuan, Yunnan, Qianhai, Ningxia, Xinjiang, Xizang
Knowledge absorption provinces	Fujian, Liaoning, Jilin, Shanxi, Anhui, Nei Mongol, Guangxi, Chongqing, Yunnan, Gansu, Qinghai, Ningxia, Xizang	Tianjin, Fujian, Liaoning, Jilin, Heilongjiang, Shanxi, Henan, Hunan, Chongqing, Sichuan, Qinghai	Hebei, Zhejiang, Fujian, Shandong, Guangdong, Heilongjiang, Anhui, Hubei, Chongqing, Guizhou, Shaanxi, Gansu

The classification of provinces into knowledge output and knowledge absorption categories reflects only the internal balance between outflow and inflow within each province. It does not imply that the absolute knowledge output of one province exceeds that of another nationwide, but rather that its relative output capacity is stronger than its absorption capacity.

**Knowledge brokers and knowledge disseminators.** Knowledge brokers function as key intermediaries that connect otherwise weakly linked regions, enabling the exchange and translation of knowledge across different communities. In phase I, apart from the knowledge-leading provinces, Shaanxi, Zhejiang, and Hubei acted as brokers. In phase II, Liaoning, Tianjin, Sichuan, and Jiangsu assumed broker roles. In phase III, Shandong, Sichuan, Guangdong, Hubei, and Zhejiang collectively served as brokers. Knowledge disseminators, in contrast, are characterized by their ability to spread comprehensive knowledge across the network. In phase I, Hubei, Guangdong, Shaanxi, and Tianjin emerged as the most effective disseminators. In phase II, Tianjin, Sichuan, and Liaoning fulfilled this role. In phase III, Guangdong, Hubei, and Zhejiang were the main disseminators.

(4) Inter-provincial correlation intensity

A robust interconnection exists among the provinces in the first echelon of each indicator. From Figs 3–5, it is evident that in phase I, Beijing maintained strong associations with Shanghai, Jiangsu, Hubei, Guangdong, Fujian, and Zhejiang. By phase II, four more provinces, namely Hunan, Anhui, Chongqing, and Shandong, joined this core network. In phase III, the number of strong relational pairs increased further. Beijing strengthened ties with additional provinces, including Henan. Simultaneously, associations between Guangdong and Heilongjiang, Hubei, Jiangsu, and Shanghai, as well as those between Shanghai and Jiangsu, Shandong, and Guangdong, intensified. These observations align with the earlier conclusion that the density of the knowledge flow network rose over time, while dependency on a few key provinces decreased.

### 4.3. The evolution of the spatial structure of the inter-provincial knowledge flow network

(1) Core–periphery analysis

Core–periphery analysis was conducted for each phase using Ucinet. Provinces with Coreness values exceeding the standard deviation of the overall Coreness were identified as core provinces. The Coreness and average values of these selected provinces are shown in Table 4.

**Table 4:** The Coreness and average Coreness of core provinces.

Phase I		Phase II		Phase III	
Core province	Coreness	Core province	Coreness	Core province	Coreness
Beijing	0.715	Beijing	0.691	Beijing	0.746
Shanghai	0.356	Shanghai	0.337	Shanghai	0.311
Jiangsu	0.321	Hubei	0.289	Jiangsu	0.291
Guangdong	0.259	Jiangsu	0.284	Hubei	0.247
Hubei	0.253	Guangdong	0.261	Guangdong	0.23
Zhejiang	0.166	Zhejiang	0.175	Tianjin	0.144
Tianjin	0.146	Tianjin	0.174		
Liaoning	0.132	Liaoning	0.157		
Mean	0.105	Mean	0.111	Mean	0.109

**Fig 11:**
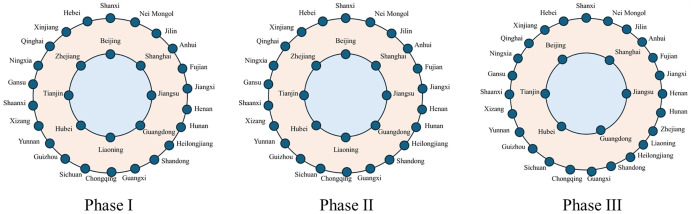
Core–periphery structures at each phase.

The corresponding core–periphery structures are illustrated in Fig 11.

The overall core–periphery configuration remained relatively stable across the first two phases. However, in phase III, Zhejiang and Liaoning were no longer part of the core. Their Coreness values dropped to 0.098 and 0.100, respectively, well below those of the remaining core provinces and significantly lower than their earlier values of [0.166, 0.175] and [0.132, 0.157]. From the perspective of provincial characteristics, most core provinces were those ranking in the first echelon of node indicators. They not only acted as the origin of knowledge spillover but also enhanced absorptive capacity. These provinces held central positions in both intermediary and comprehensive knowledge flows, reflecting their role as China’s educational and research hubs.

The densities of the core and periphery regions across phases are presented in Table 5. Although core-region density increased overall, the rate of growth was lower than that of the periphery. Stronger connectivity between the core and periphery coincided with a rise in overall network density, despite a decrease in network centralization. This suggests that reliance on the core diminished over time, and the disparity between provinces in terms of network significance gradually narrowed.

**Table 5:** Density of Core-Periphery in each phase.

	Phase I		Phase II		Phase III	
Core	Edge	Core	Edge	Core	Edge
Core	25.018	4.663	24.911	4.924	60.233	14.200
Edge	3.560	0.727	3.614	0.802	8.773	2.498

(2) Network community structure

**Fig 12:**
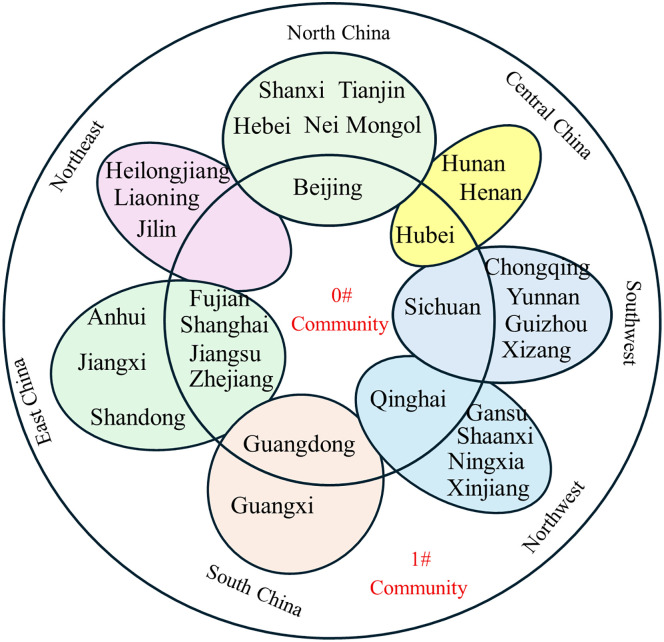
Community affiliation and geographical division of provinces at Phase I.

**Fig 13:**
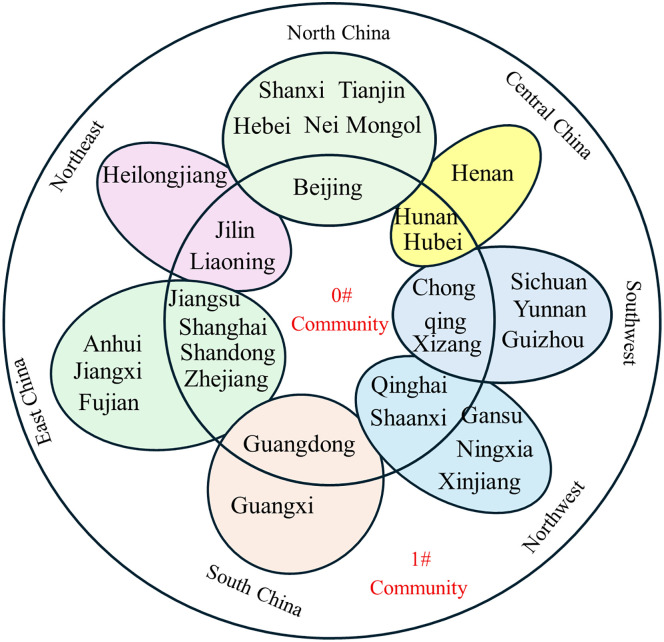
Community affiliation and geographical division of provinces at Phase II.

**Fig 14:**
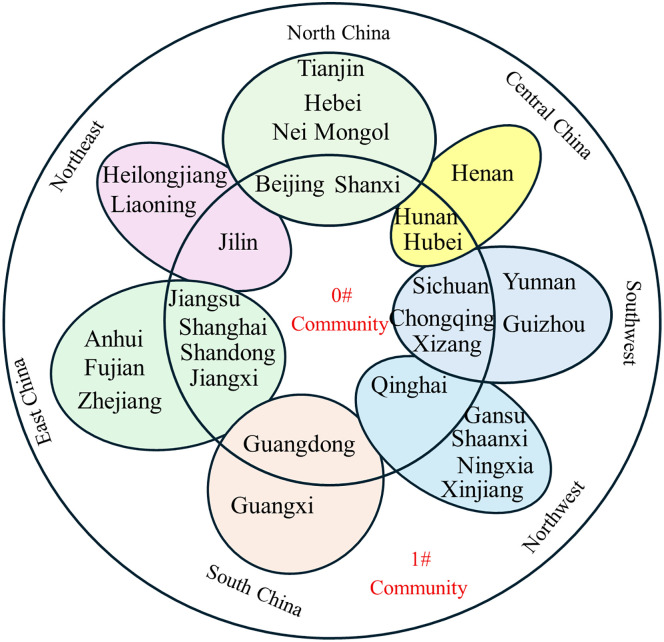
Community affiliation and geographical division of provinces at Phase III.

**Fig 15:**
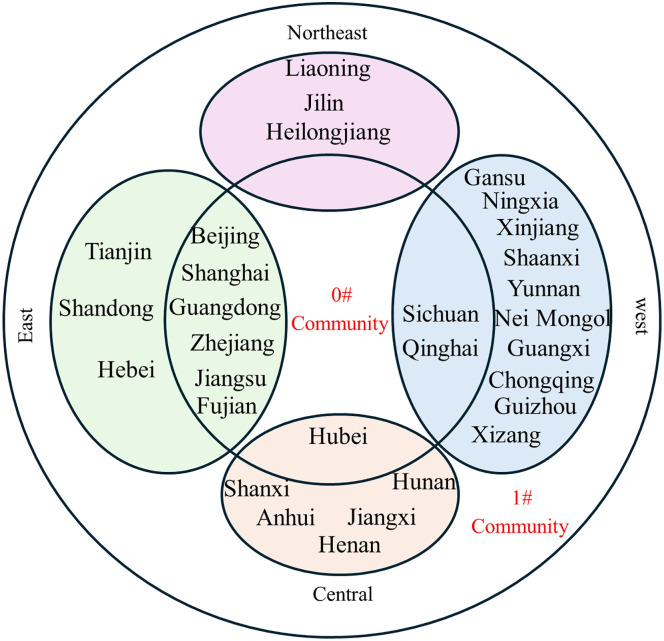
Community affiliation and economic division of provinces at Phase I.

**Fig 16:**
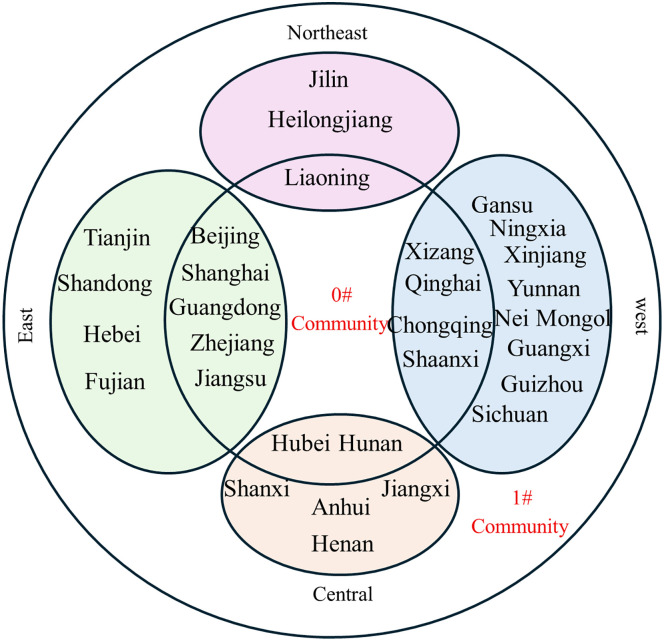
Community affiliation and economic division of provinces at Phase II.

**Fig 17:**
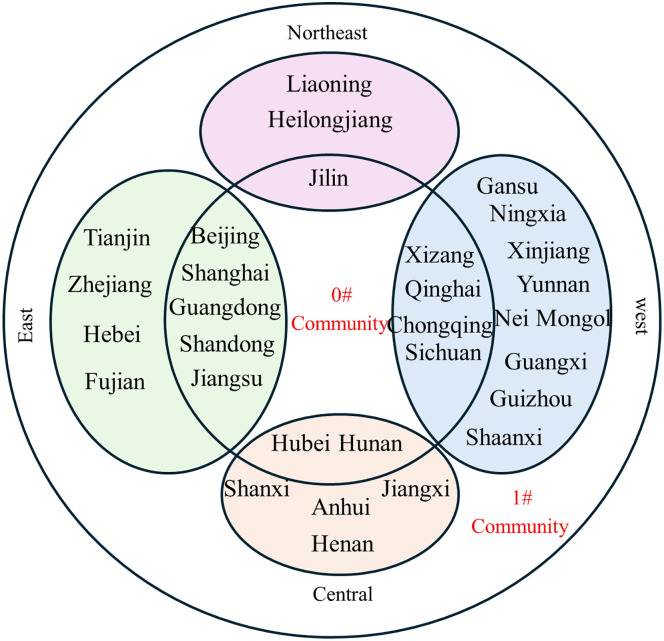
Community affiliation and economic division of provinces at Phase III.

The partitioning of the inter-provincial knowledge flow network, as determined by the Louvain algorithm, is shown in Fig 12-17.

At each Phase, the network was divided into two major communities. While membership shifted slightly between phases, no tightly clustered subgroups emerged. In phase I, Community 0^#^—comprising Beijing, Shanghai, Jiangsu, Guangdong, and Hubei—occupied central positions in the network and were characterized by abundant, high-quality knowledge resources. In phases II and III, the membership of Community 0^#^ expanded, indicating that more provinces gravitated toward knowledge-rich regions to strengthen their own knowledge bases. The distribution of provinces across communities showed limited correlation with geographical proximity or economic grouping. For example, Jilin and Guangdong belonged to the same community despite their geographical distance, while provinces with stark economic disparities, such as Guangdong and Qinghai, were consistently members of Community 0^#^. This divergence is particularly evident when comparing provincial GDP. For instance, Guangdong’s average GDP across the three phases was 51,587.56, 81,726.86, and 121,518.68 billion yuan, respectively, whereas Qinghai’s was far lower at 1,619.35, 2,560.04, and 3,345.41 billion yuan. Despite these disparities, both provinces were part of Community 0^#^. This indicates that knowledge flow is shaped primarily by the recognition of knowledge quality rather than geographical or economic proximity, facilitating the dissemination of high-value knowledge across regions.

### 4.4. Evolution of knowledge characteristics in the inter-provincial knowledge flow network

The indicator values of knowledge characteristics for each province at each phase were calculated based on the defined indexes and methods (Table 2 in Appendix).

From the horizontal comparison across phases, the variation in the breadth of knowledge inflow and outflow is relatively limited, suggesting that the number of research topics of concern to each province has remained largely stable. By contrast, the depth of knowledge inflow and outflow exhibits pronounced variation across provinces. For most provinces, depth increased steadily, particularly in phases II and III, when improvements became more substantial. However, reductions in knowledge depth were observed in a few provinces, including Fujian, Liaoning, Jilin, Jiangxi, Nei Mongol, and Xinjiang, and these reductions were confined to the depth of knowledge outflow.

From the longitudinal perspective, several patterns emerge. In terms of knowledge outflow breadth, Beijing, Shanghai, Jiangsu, Zhejiang, Guangdong, Hubei, and Shaanxi show greater diversity in their knowledge dissemination. Regarding inflow breadth, most provinces outside western China absorb more than ten categories of knowledge. In many provinces, the breadth of absorption exceeds that of output, indicating both an expansion of provincial knowledge reserves and an enhanced capacity to integrate diverse knowledge resources for innovation. For knowledge outflow depth, Beijing, Shanghai, Jiangsu, Zhejiang, Guangdong, and Hubei consistently exhibit higher levels, each following an upward trajectory. Except for Zhejiang, these provinces also form the core region in phase III. In contrast, Tianjin and Fujian, despite demonstrating relatively high knowledge outflow depth in the first phase, followed a downward trajectory in subsequent phases. For knowledge inflow depth, Beijing, Shanghai, Jiangsu, Guangdong, and Hubei again ranked highest across phases, highlighting their strong absorptive capacity. From a developmental standpoint, Chongqing, Shaanxi, Shandong, and Zhejiang have experienced rapid growth in knowledge inflow depth, indicating that these provinces are not only broadening their knowledge acquisition but also intensifying efforts to explore and exploit specialized knowledge domains.

## 5. Conclusions and suggestions

### 5.1. Conclusions

This study investigates the characteristics and evolutionary patterns of the inter-provincial knowledge flow network in China by analyzing citation data from core Chinese journals spanning the period 2009–2023. Using social network analysis, we examine three dimensions: topological structure, node characteristics, and spatial configuration. We further assess the breadth and depth of inter-provincial knowledge diffusion by leveraging the “CLC Number” as an indicator. The main conclusions are as follows: (1) From the perspective of topological evolution, the frequency of inter-provincial knowledge flow has substantially increased; (2) Regarding node features, both knowledge absorption capacity (measured by in-degree centrality) and knowledge diffusion capacity (measured by out-degree centrality) exhibit a consistent upward trend; (3) From the perspective of spatial structure, the developmental gap between core provinces and peripheral provinces has been gradually narrowing, whereas the distribution of cross-community provinces does not exhibit significant correlation with either geographical proximity or economic clustering; (4) In terms of the evolution of knowledge characteristics, both the depth of knowledge inflow and outflow have increased significantly, indicating that provinces are becoming increasingly specialized and are generating higher-value research outputs.

### 5.2. Discussion

This paper exhibits the following characteristics in its investigation of the evolution and features of China’s inter-provincial knowledge flow network: Firstly, it constructs a network based on citation linkages among literature from Chinese provinces, reflecting how knowledge innovation actors in each province recognize the quality and characteristics of knowledge, as well as indicating the relative significance of each province in advancing China’s knowledge innovation development. Secondly, this paper not only employs social network analysis to examine the structural characteristics of the provincial knowledge flow network, but also measures the breadth and depth of inter-provincial knowledge flows by leveraging Chinese Library Classification codes, thereby extending the existing literature through methodological innovation. Finally, although this paper focuses on Chinese literature as the research data, the data processing and methodological framework employed are broadly applicable, offering valuable insights for future studies on knowledge flow networks in other national contexts.

Consistent with prior studies, this research further confirms that knowledge interaction among nodes within the knowledge flow network is increasingly frequent [64,65], network centralization progressively diminishes [66], the small-world characteristic of the network becomes more pronounced, and the average path length between nodes steadily decreases [67].

However, by further examining the structural characteristics of the knowledge flow network and the inherent features of knowledge, this study identifies three key differences. Existing research generally indicates that economic, geographical, and knowledge-based similarities play a significant role in shaping the formation and evolution of knowledge flow networks [13], as well as in strengthening the ties between network nodes. However, the knowledge flow network based on citations does not exhibit clustering according to geographical proximity or economic similarity. Instead, connections are driven by knowledge quality and innovation capacity: provinces with higher scores generate more impactful research outcomes and attract stronger citation networks. Secondly, in studies focusing on collaboration or knowledge transfer, the contribution of knowledge-poor provinces to regional knowledge innovation is often obscured by mechanisms such as link preference and the Matthew effect [1,68], making it difficult to recognize their role. In this study, we observe that as the knowledge flow network evolves through the second and third phases, provinces initially lacking in knowledge gradually gain recognition from other provinces, leading to an increase in both the breadth and depth of knowledge dissemination. This indicates that the structure of the knowledge flow network, derived from knowledge citations, is better suited to revealing the position and role of knowledge-poor nodes within the network. This has practical implications for identifying disparities between resource-rich and resource-poor nodes and supports efforts to reduce this gap. Thirdly, in existing studies on knowledge flows between provinces or cities, major urban centers frequently serve as key knowledge intermediaries—for instance, Beijing, Shanghai, and Nanjing in China [26,27], and New York and London internationally [29]. However, the analysis of this study reveals that certain provinces not ranked at the top also serve as intermediaries — for instance, Liaoning in the second phase and Sichuan in both the second and third phases. This phenomenon is attributable to the fact that knowledge citations in this context prioritize knowledge quality over other potential influencing factors. Finally, in networks characterized by collaboration and knowledge transfer, multiple core-periphery structures have emerged, featuring key nodes positioned at the core and peripheral nodes distributed across less central regions. Notable examples include the Beijing-Tianjin-Hebei knowledge flow network, which is centered on Beijing and Tianjin and extends outward to surrounding cities, as well as the Jiangsu-Zhejiang-Shanghai knowledge flow network, anchored by Shanghai, Hangzhou, and Nanjing, serving as primary hubs of knowledge diffusion [28]. This is because geographical proximity frequently influences the structure of collaborative networks and facilitates knowledge transfer [69]. However, such structures are not statistically significant in the knowledge flow networks at each phase based on citation patterns. The community structure remains unstable, and the core-periphery pattern gradually blurs, further indicating that geographical location and economic status exert only limited influence on the network topology within citation-based knowledge flow networks.

Based on the above discussions, the following countermeasures are proposed:

(1) Strengthen in-depth tracking of high-quality research outcomes.

High-quality, high-impact research outcomes should be tracked systematically to better evaluate their long-term value and influence. In citation-based knowledge flow networks, provinces occupying central positions generate large volumes of high-quality knowledge and sustain their output by both assimilating high-quality knowledge (high in-degree centrality) and drawing from diverse sources (high betweenness centrality). Tracking the innovation achievements and research trajectories of these key nodes, and promoting valuable research directions, can help peripheral provinces enhance their knowledge innovation capacity more effectively.

(2) Develop tailored regional research and innovation strategies.

Innovation strategies should be aligned with the roles of provinces within the knowledge flow network rather than applying uniform measures. Knowledge-leading provinces should maintain steady growth in R&D investment, strengthen organizational flexibility, and enhance global leadership through international collaboration and strategic partnerships. Knowledge-output and knowledge-absorbing provinces should establish inter-provincial platforms for knowledge exchange, leverage differences in resource endowments, and promote interdisciplinary collaboration and regional integration. Provinces lagging in all indicators should pursue a dual strategy: first, learning from the practices through which knowledge-leading provinces strengthen their research and innovation; second, identifying unique regional advantages, bridging structural holes in the network, and improving knowledge acquisition efficiency by emphasizing in-depth exploration of niche knowledge domains.

(3) Formulate knowledge innovation strategies tailored to regional characteristics

Policymakers should retain a macro-level view of disciplinary development while encouraging differentiated regional contributions. On one hand, support should be directed toward the stable advancement of foundational disciplines. On the other, the cultural and resource characteristics of regions should be leveraged to foster a diverse knowledge ecosystem. For example, research on ethnic minority issues is concentrated in Yunnan and Guizhou, marine studies in coastal provinces, and heavy industry in the northeast. This spatial heterogeneity enriches national knowledge reserves and reflects the principles of knowledge base theory, which holds that diversified knowledge development and effective circulation stimulate new knowledge creation and technological innovation. Thus, even provinces that are not strong in output quality or network centrality can make meaningful contributions by preserving and enhancing the uniqueness of their knowledge. Diversity in knowledge reserves remains a fundamental guarantee of national innovation capacity.

Finally, several limitations should be acknowledged. This study constructed the national inter-provincial knowledge flow network solely from citation relationships of Chinese-language literature, using the top 500 most-cited papers each year. Hong Kong, Macao, and Taiwan were excluded, despite their strong contributions in foreign-language research. Future research should expand data sources to include international and English-language publications, as well as broader corpora, to construct a more comprehensive picture of knowledge flows and generate more robust findings.

## Supporting information

S1 File Appendices. (DOCX)

S2 File Raw data. (XLSX)

S3 File Citation relationship matrix. (XLSX)
