# Supplementary material for: Uncovering the characteristics and evolution of inter-provincial knowledge flow in China through Chinese literature citations
Source: PLoS One. 2025 Nov 12;20(11):e0336249. doi: 10.1371/journal.pone.0336249 (PMC12611130; doi:10.1371/journal.pone.0336249)
Supplement: S1 File — (DOCX) [file pone.0336249.s001.docx]

**Appendices:**

**Table 1 The O-I index of each province at each phase**

| Provinces | *O-I* | | | Provinces | *O-I* | | |
| --- | --- | --- | --- | --- | --- | --- | --- |
|  | Phase I | Phase II | Phase III |  | Phase I | Phase II | Phase III |
| Beijing | **0.0186** | -0.0186 | -0.0173 | Henan | 0.0000 | -0.1429 | **0.0199** |
| Tianjin | **0.0455** | -0.0800 | **0.0186** | Hubei | **0.1304** | 0.0000 | -0.0185 |
| Hebei | **0.0394** | **0.1206** | -0.1304 | Hunan | **0.0500** | -0.0953 | **0.0984** |
| Shanghai | **0.0400** | -0.0199 | 0.0000 | Nei Mongol | -0.5012 | **0.0394** | **0.0766** |
| Jiangsu | **0.0385** | **0.0400** | 0.0000 | Guangxi | -0.0667 | **0.2182** | **0.0815** |
| Zhejiang | **0.0236** | **0.0417** | -0.0185 | Chongqing | -0.0260 | -0.0455 | -0.0800 |
| Fujian | -0.0240 | -0.0736 | -0.0833 | Sichuan | 0.0000 | -0.0615 | **0.0185** |
| Shandong | **0.0260** | **0.0526** | -0.0800 | Guizhou | **0.3757** | **1.0000** | -0.0260 |
| Guangdong | **0.0455** | **0.0000** | -0.0185 | Yunnan | -0.0470 | **0.1433** | **0.0400** |
| Liaoning | -0.0500 | -0.0635 | **0.0186** | Shaanxi | **0.0219** | **0.0000** | -0.0385 |
| Jilin | -0.0327 | -0.0299 | **0.0615** | Gansu | -0.0909 | **0.0000** | -0.1251 |
| Heilongjiang | **0.1348** | -0.0526 | -0.0186 | Qinghai | -1.0000 | -1.0000 | **0.1671** |
| Shanxi | -0.4282 | -0.0340 | 0.0000 | Ningxia | -0.2000 | **0.3333** | **0.1053** |
| Anhui | -0.1053 | **0.0455** | -0.0186 | Xinjiang | **0.1206** | **0.0914** | **0.0186** |
| Jiangxi | 0.0000 | **0.0000** | **0.0953** | Xizang | -1.0000 | **1.0000** | **1.0000** |

**Table 2 the indicator values of knowledge characteristics of provinces**

| Provinces | | Breadth of knowledge outflow | | | Breadth of knowledge inflow | | | Depth Of Knowledge Outflow | | | Depth Of Knowledge Inflow | | |
| --- | --- | --- | --- | --- | --- | --- | --- | --- | --- | --- | --- | --- | --- |
|  |  | Phase I | Phase II | Phase III | Phase I | Phase II | Phase III | Phase I | Phase II | Phase III | Phase I | Phase II | Phase III |
| Eastern  Region | Beijing | 14 | 14 | 13 | 16 | 16 | 15 | 4.534 | 5.283 | 9.746 | 4.015 | 4.167 | 6.504 |
|  | Tianjin | 7 | 7 | 7 | 8 | 10 | 11 | 4.064 | 3.561 | 3.762 | 3.695 | 3.172 | 3.800 |
|  | Hebei | 4 | 5 | 5 | 8 | 7 | 7 | 0.683 | 1.295 | 1.736 | 1.561 | 1.060 | 1.548 |
|  | Shanghai | 11 | 11 | 12 | 14 | 12 | 11 | 3.888 | 3.035 | 4.548 | 4.832 | 3.611 | 5.449 |
|  | Jiangsu | 15 | 13 | 9 | 15 | 14 | 16 | 2.796 | 2.432 | 5.600 | 2.515 | 2.846 | 4.865 |
|  | Zhejiang | 13 | 9 | 8 | 14 | 11 | 13 | 2.035 | 2.404 | 4.121 | 2.566 | 2.610 | 3.593 |
|  | Fujian | 7 | 6 | 7 | 11 | 9 | 10 | 4.427 | 3.829 | 2.080 | 3.392 | 4.053 | 3.617 |
|  | Shandong | 8 | 7 | 9 | 11 | 9 | 12 | 1.910 | 2.104 | 3.186 | 2.101 | 2.527 | 4.000 |
|  | Guangdong | 12 | 12 | 11 | 12 | 14 | 12 | 3.976 | 3.520 | 4.270 | 3.999 | 3.693 | 4.585 |
| Northeast  Region | Liaoning | 8 | 8 | 9 | 9 | 11 | 11 | 2.888 | 3.141 | 1.893 | 1.761 | 3.395 | 3.390 |
|  | Ji Lin | 6 | 6 | 5 | 9 | 9 | 11 | 1.939 | 1.369 | 1.567 | 2.359 | 1.657 | 2.730 |
|  | Heilongjiang | 6 | 6 | 7 | 12 | 10 | 9 | 1.406 | 1.351 | 2.528 | 1.513 | 1.661 | 1.731 |
| Central  Region | Shanxi | 4 | 4 | 4 | 7 | 7 | 9 | 1.000 | 1.900 | 2.098 | 1.172 | 1.238 | 1.983 |
|  | Anhui | 5 | 7 | 5 | 14 | 10 | 10 | 1.801 | 2.354 | 3.750 | 1.486 | 2.240 | 2.446 |
|  | Jiangxi | 4 | 6 | 4 | 12 | 13 | 10 | 2.706 | 1.574 | 1.724 | 1.618 | 1.681 | 2.100 |
|  | Henan | 7 | 5 | 7 | 9 | 8 | 11 | 1.522 | 1.868 | 2.764 | 1.510 | 1.428 | 2.369 |
|  | Hubei | 14 | 10 | 9 | 15 | 12 | 12 | 3.234 | 3.267 | 4.792 | 4.181 | 3.177 | 4.551 |
|  | Hunan | 8 | 8 | 7 | 12 | 11 | 13 | 2.811 | 2.180 | 2.504 | 2.635 | 2.304 | 3.203 |
| Western  Region | Nei Mongol | 1 | 2 | 3 | 7 | 5 | 8 | 1.000 | 1.250 | 0.926 | 0.591 | 1.051 | 1.317 |
|  | Guangxi | 3 | 2 | 5 | 9 | 7 | 8 | 0.702 | 1.667 | 1.304 | 1.385 | 1.660 | 2.047 |
|  | Chongqing | 7 | 6 | 5 | 8 | 8 | 11 | 1.942 | 1.982 | 2.487 | 4.382 | 2.068 | 3.437 |
|  | Sichuan | 7 | 8 | 6 | 10 | 9 | 14 | 1.868 | 2.766 | 3.033 | 2.534 | 3.251 | 3.211 |
|  | Guizhou | 4 | 0 | 6 | 4 | 6 | 6 | 0.750 | 0.000 | 1.817 | 1.417 | 1.815 | 1.846 |
|  | Yunnan | 4 | 4 | 2 | 8 | 7 | 10 | 1.217 | 1.239 | 2.188 | 1.029 | 1.742 | 1.715 |
|  | Shaanxi | 9 | 8 | 10 | 14 | 13 | 12 | 2.995 | 2.477 | 3.330 | 2.770 | 1.927 | 3.685 |
|  | Gansu | 4 | 3 | 6 | 9 | 9 | 11 | 0.667 | 0.756 | 1.890 | 1.285 | 1.196 | 2.292 |
|  | Qinghai | 0 | 2 | 2 | 3 | 0 | 6 | 0.000 | 0.500 | 0.500 | 0.500 | 0.000 | 1.226 |
|  | Ningxia | 3 | 2 | 2 | 6 | 4 | 6 | 0.556 | 0.875 | 1.382 | 0.571 | 1.250 | 0.742 |
|  | Xinjiang | 3 | 3 | 5 | 6 | 8 | 8 | 2.000 | 1.556 | 1.521 | 1.402 | 1.639 | 1.633 |
|  | Xizang | 0 | 0 | 0 | 2 | 2 | 2 | 0.000 | 0.000 | 0.000 | 0.500 | 0.500 | 1.500 |
